# Gene map of large yellow croaker (*Larimichthys crocea*) provides insights into teleost genome evolution and conserved regions associated with growth

**DOI:** 10.1038/srep18661

**Published:** 2015-12-22

**Authors:** Shijun Xiao, Panpan Wang, Yan Zhang, Lujing Fang, Yang Liu, Jiong-Tang Li, Zhi-Yong Wang

**Affiliations:** 1 Key Laboratory of Healthy Mariculture for the East China Sea, Ministry of Agriculture, Fisheries College, Jimei University, Yindou Road, Xiamen, P.R. China; 2 CAFS Key Laboratory of Aquatic Genomics and Beijing Key Laboratory of Fishery Biotechnology, Centre for Applied Aquatic Genomics, Chinese Academy of Fishery Sciences, Beijing, 100141, P.R. China

## Abstract

The genetic map of a species is essential for its whole genome assembly and can be applied to the mapping of important traits. In this study, we performed RNA-seq for a family of large yellow croakers (*Larimichthys crocea*) and constructed a high-density genetic map. In this map, 24 linkage groups comprised 3,448 polymorphic SNP markers. Approximately 72.4% (2,495) of the markers were located in protein-coding regions. Comparison of the croaker genome with those of five model fish species revealed that the croaker genome structure was closer to that of the medaka than to the remaining four genomes. Because the medaka genome preserves the teleost ancestral karyotype, this result indicated that the croaker genome might also maintain the teleost ancestral genome structure. The analysis also revealed different genome rearrangements across teleosts. QTL mapping and association analysis consistently identified growth-related QTL regions and associated genes. Orthologs of the associated genes in other species were demonstrated to regulate development, indicating that these genes might regulate development and growth in croaker. This gene map will enable us to construct the croaker genome for comparative studies and to provide an important resource for selective breeding of croaker.

Teleosts are widely believed to have undergone three rounds of whole genome duplication (WGD), one more round than mammals have undergone^1^,^2^. The third round of WGD occurrred 370 million years ago (MYA)^3^. In a relatively short period of ~50 MYA after the WGD event, the common ancestor of most teleosts underwent eight major inter-chromosomal rearrangements, leading to an ancestral karyotype consisting of 24 chromosomes^4^. Intriguingly, the medaka (*Oryzias latipes*) genome preserved the ancestral karyotype for more than 300 MYA^5^. Although many teleost genomes have been published, comparative analysis revealed that they underwent inter-chromosomal rearrangements after speciation from a common ancestor^6^,^7^. Thus, these species have more or fewer chromosomes than the medaka. Whether other species preserves the ancestral karyotype, as the medaka has, remains unknown.

Large yellow croaker (hereafter referred to as ‘croaker’), *Larimichthys crocea*, is a member of the Sciaenidae family, which includes more than 270 species in the order Perciformes. However, high-density genetic mapping and chromosome comparison of Sciaenidae has rarely been reported. Previous comparative genomic analysis based on the sequence conservation among orthologs and protein-coding sequence similarity revealed that the stickleback (*Gasterosteus aculeatus*, order Gasterosteiformes) is more closely related to the croaker than other teleosts, for instance, the medaka and tetraodon (*Tetraodon nigroviridis*)^8^,^9^. However, the chromosome number of the croaker (24) is equal to that of the medaka (24)^5^,^10^ and is different from those of the stickleback (21)^11^ and tetraodon (21)^12^, which indicates that dramatic and different genome rearrangements occurred after these species diverged. Because no genetic map is available to anchor the genomic sequence of the croaker, it is difficult to perform a chromosome-wide comparison among the croaker and other teleosts. Thus, the construction of a high density genetic map is desirable for genome assembly and downstream genome-wide comparison.

In this study, we performed transcriptome-sequencing for all progeny of a mapping family of croakers. After genotyping, we constructed a high-density genetic map, the markers of which were mainly protein-coding. We used coding markers on the croaker map to examine the synteny between the croaker and other five fish species, including the stickleback, tetraodon, zebrafish (*Danio rerio*), medaka and spotted gar (*Lepisosteus oculatus*). Finally, using the QTL analysis and the association study, we identified candidate genes related to the growth traits of the croaker.

## Results

### SNP discovery using genome sequencing and RNA-seq

The genome size of the croaker was estimated to be ~725 Mb^10^,^13^. Genome sequencing was performed for each parent by sequencing paired-end DNA libraries with an insert size of 300 bp on an Illumina HiSeq2000 platform. We sequenced 33.8 Gb of data for the male parent, equivalent to 45 × genome coverage. The genome assembly of the male parent was 720 Mb in length with a scaffold N50 size of 356 bp. Because only one library was sequenced, many scaffolds of the assembly were small. However, the assembly covered 99.3% of the estimated genome size, greater than the public genome assemblies of female croakers, which covered only 644 Mb^8^ and 679 Mb^9^, respectively. All scaffold sequences are accessible at http://lycgenomics.jmu.edu.cn/maleGenome/ and were used as the reference genome for further genotyping.

We also sequenced 25.4 Gb of data for the female parent with almost 33.8 × genome coverage. We performed alignments for all sequences represented in two parents, using the same criteria (see Methods) to group the sequences into distinct loci. The grouping resulted in 505 Mb distinct loci that were shared by both parents. Considering that the average sequencing depth in each parent was over 30 × , these shared distinct loci were represented as high-quality reference sites. We used stringent criteria to identify high-quality SNPs from the whole genome sequencing results of the parents. Among the reference sites, more than 8 million loci contained one biallelic SNP in at least one parent.

For each offspring, the high-quality RNA-seq data with an average of 13.9 million reads were obtained. We aligned the RNA-seq reads against the reference sites for genotyping. A total of 23,790 polymorphic markers were genotyped in at least 80% of individuals and were heterozygous in at least one parent. For these polymorphic markers, a high depth (49 × depth on average) was sequenced per offspring (Supplementary Figure 1), suggesting that these polymorphic markers were of high quality.

### High-resolution linkage mapping

A chi-square test identified 8,311 markers that conformed to expected Mendelian ratios (p-value > 0.05). Because JoinMap allows a limited number of markers^14^, the marker number was reduced; for one short genomic sequence (<500 bp in length) including multiple markers, only one marker with the highest coverage among all individuals was selected to represent this sequence and was included in the following analysis. At the LOD threshold of 5.0, JoinMap assigned 3,448 markers to 24 linkage groups, consistent with the haploid chromosome number of the croaker^10^ (see details in Supplementary Figure 2 and Supplementary Table 1). As shown in Table 1, the total map length obtained was 2,632 cM with an average marker interval of 0.76 cM. LG17, the longest croaker linkage group, comprised 180 markers in 158.5 cM; and LG2, the shortest linkage group, comprised 90 markers in 67.8 cM. The 3,448 markers were distributed among 2,608 genomic sequences, 99.5% of which were over 200 bp in length (Supplementary Figure 3), suggesting that these sequences were long enough to design primers for further validation and selective breeding.

### Estimation of map validity

The assignment of the mapped markers to genomic sequences provided a test of map validity. For 601 long genomic sequences including at least two markers, only eleven sequences were assigned to different linkage groups, indicating that the error rate of this map was very low, at only 1.8% (11 out of 601).

To validate reference sequences with potentially inaccurate SNP markers, the eleven sequences were PCR amplified in four randomly selected individuals, including father and three offspring (sample 44, sample 72 and sample 77). The primers are listed in Supplementary Table 2. The successful amplifications (Supplementary Figure 4) of all samples validated the assembly of reference sequences. We also estimated the accuracy of variant calling using genotypes obtained from Sanger sequencing as reference. A total of 36 amplifications from nine randomly selected markers across four samples were subjected to Sanger sequencing. The comparison between SNPs obtained from Sanger sequencing and from RNA-sequencing showed that 35 markers in four samples identified by RNA-seq were successfully detected by conventional sequencing, except one marker (C39669009_238) in father (Supplementary Figure 5); thus, the true positive rate was estimated to be 97.2% (35 out of 36). The high consistence was possibly due to the high sequencing depth obtained for each individual.

### Comparative genomics

Because the mapping markers were identified through transcriptome-sequencing, they represent a valuable resource for future comparative genome analysis. To investigate genome rearrangements across teleosts, we performed Blastx^15^ searches using croaker markers against proteins from the stickleback, tetraodon, zebrafish, medaka and spotted gar. We identified 1,660, 1,728, 1,790, 1,809 and 1,715 genomic sequences (covering 2,145, 2,232, 2,246, 2,284, and 2,162 markers, respectively) having significant gene hits against the medaka, stickleback, tetraodon, zebrafish and spotted gar genes, respectively. We retained the most significant gene hit for each marker in the following analysis. Homolog search identified 1,941 croaker genomic sequences (with 2,459 markers) having putative orthologs in at least one of the other five genomes. Alignment against NCBI nr database identified another 24 coding genomic sequences (with 36 markers). Detailed homolog relationships between the croaker sequences and proteins from other species are summarized in Supplementary Table 3. With these coding markers distributed over the linkage groups, we were able to perform comparative genomic analyses. We constructed Oxford grids that aligned croaker coding markers in their genomic order along the horizontal axis and plotted the position of each ortholog against the other genomes, which were displayed along the vertical axis.

**Figure 1:**
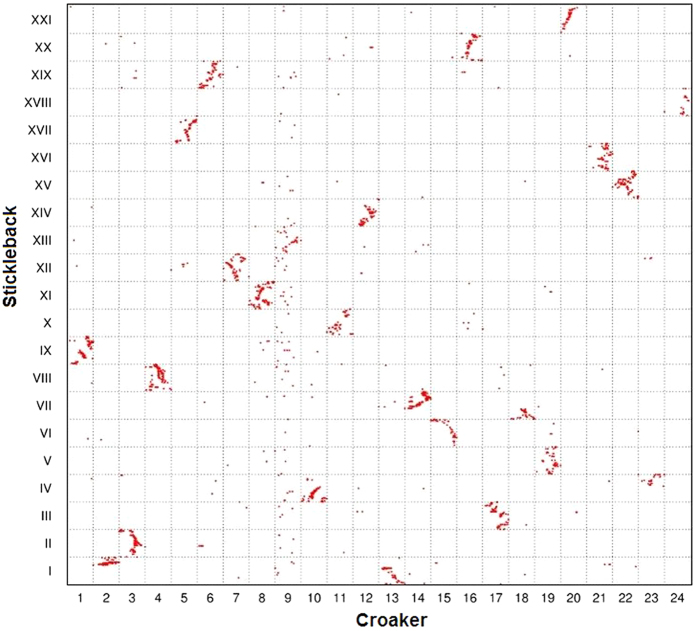
Genomic comparisons between large yellow croaker and stickleback. Each red point represents the position of an orthologous gene pair in the corresponding croaker and stickleback genomes. Synteny comparison revealed that three stickleback chromosomes exhibited 1:2 syntenic correspondences to croaker linkage groups.

First, the Oxford grid between the croaker and stickleback (Fig. 1) revealed that 18 of the 24 croaker chromosomes (1, 3, 4, 5, 6, 7, 8, 9, 11, 12, 15, 16,17, 19, 20, 21, 22, and 24) were in relatively conserved synteny with stickleback chromosomes (IX, II, VIII, XVII, XIX, XII, XI, XIII, X, XIV, VI, XX, III, V, XXI, XVI, XV and XVIII), respectively. Except the 1:1 correspondence of chromosomes in the two species, three stickleback chromosomes were found to have syntenic blocks with two croaker chromosomes. Chromosome I of stickleback orthologs occurred in two croaker linkage groups, LG2 and LG13. Likewise, stickleback chromosome IV proved co-orthologous to croaker LG10 and LG23. Croaker genes that mapped to LG14 and LG18 had orthologs that were distributed between two different portions of stickleback chromosome VII. The 1:2 synteny between three stickleback chromosomes and six croaker linkage groups indicated the chromosome fusion events in stickleback.

**Figure 2:**
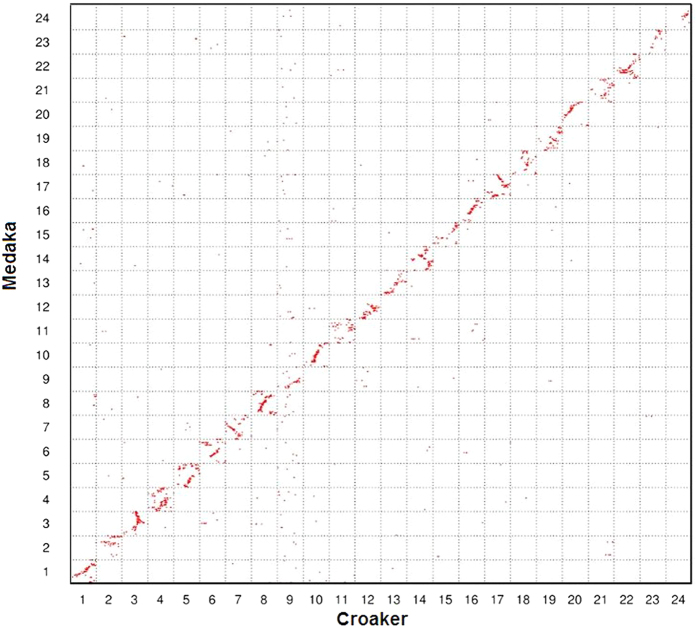
Genomic comparisons between large yellow croaker and medaka. Each red point in the Oxford grid represents the position of an orthologous gene pair in the corresponding croaker and medaka genomes. Almost all croaker and medaka chromosomes exhibited 1:1 correspondence.

Second, in the grid, 2,178 croaker markers were arranged in their genomic order along the horizontal axis, and the position of each medaka ortholog was plotted against the medaka genome, which was displayed along the vertical axis (Fig. 2). The major markers in the croaker chromosomes tended to be represented in just one medaka chromosome. We did not observe a major genome rearrangement between the medaka and croaker. Although some markers in croaker LG9 were distributed among several medaka chromosomes, most markers in croaker LG9 were represented on medaka chromosome 9.

**Figure 3:**
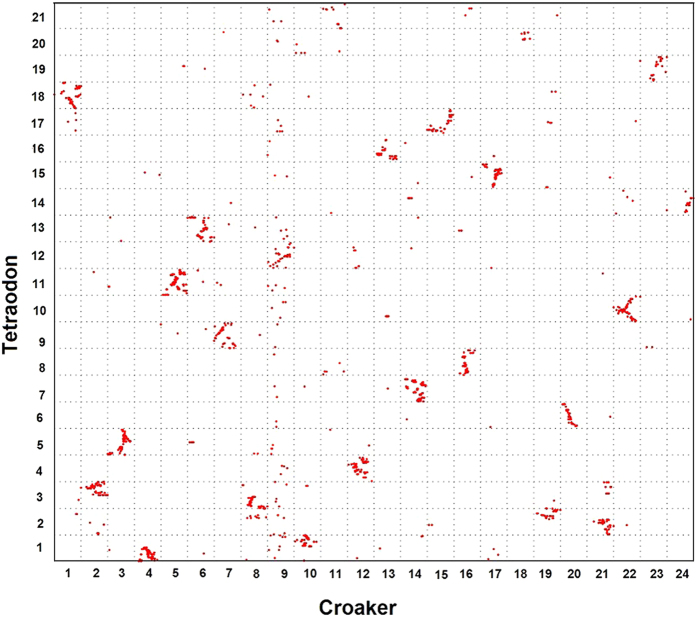
Genomic comparisons between large yellow croaker and tetraodon. In tetraodon, three chromosomes exhibited 1:2 syntenic correspondences to croaker linkage groups.

Third, a comparison of the croaker gene map with the tetraodon genome revealed that three chromosome fusions occurred after the speciation between the croaker and tetraodon. The Oxford grid between the croaker and tetraodon (Fig. 3) revealed that 18 of the 24 croaker chromosomes (1, 3, 5, 6, 7, 9, 11, 12, 13, 14, 15, 16, 17, 18, 20, 22, 23, and 24) exhibited relatively conserved synteny with tetraodon chromosomes (18, 5, 11, 13, 9, 12, 21, 4, 16, 7, 17, 8, 15, 20, 6, 10, 19, and 14), respectively. Each of the three tetraodon chromosomes was found to have syntenic blocks with two croaker chromosomes. The orthologs in tetraodon chromosome 1 were found on croaker LG4 and LG10. Likewise, tetraodon chromosome 2 tended to be found in croaker LG19 and LG21. The markers located on croaker LG2 and LG8 had tetraodon orthologs along chromosome 3.

**Figure 4:**
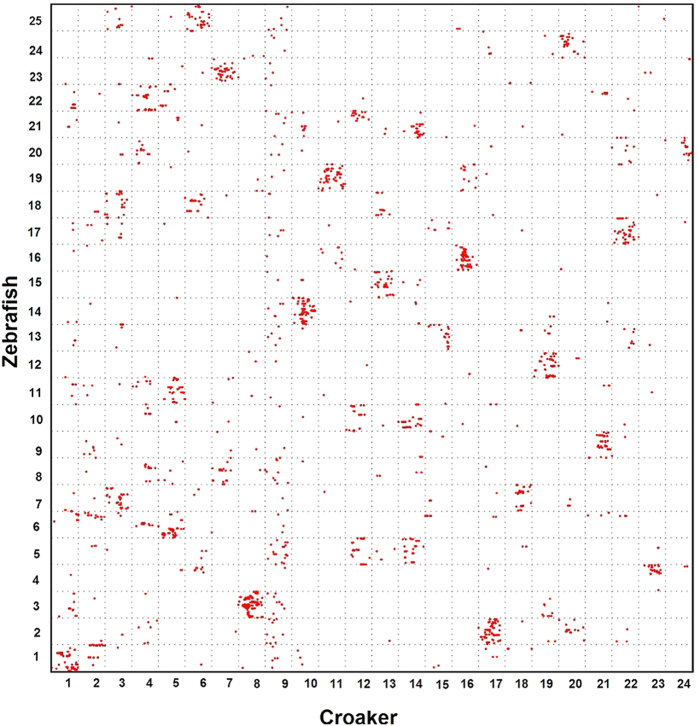
Genomic comparisons between large yellow croaker and zebrafish. Genome rearrangements in zebrafish are more complex than those in other teleosts.

Fourth, the comparison analysis showed that more complex genome rearrangements were present in the zebrafish than in the medaka, stickleback or tetraodon (Fig. 4). Although many zebrafish chromosomes tended to be represented on just one croaker chromosome, we observed that some chromosomes were orthologous to multiple croaker linkage groups. For instance, each of chromosomes 7, 10, 18, 20 and 21 tended to fall in two croaker linkage groups. Furthermore, zebrafish genes in chromosomes of 5 and 8 had orthologs that were distributed broadly among three croaker chromosomes (LG9, LG12 and LG14; and LG4, LG7 and LG9).

**Figure 5:**
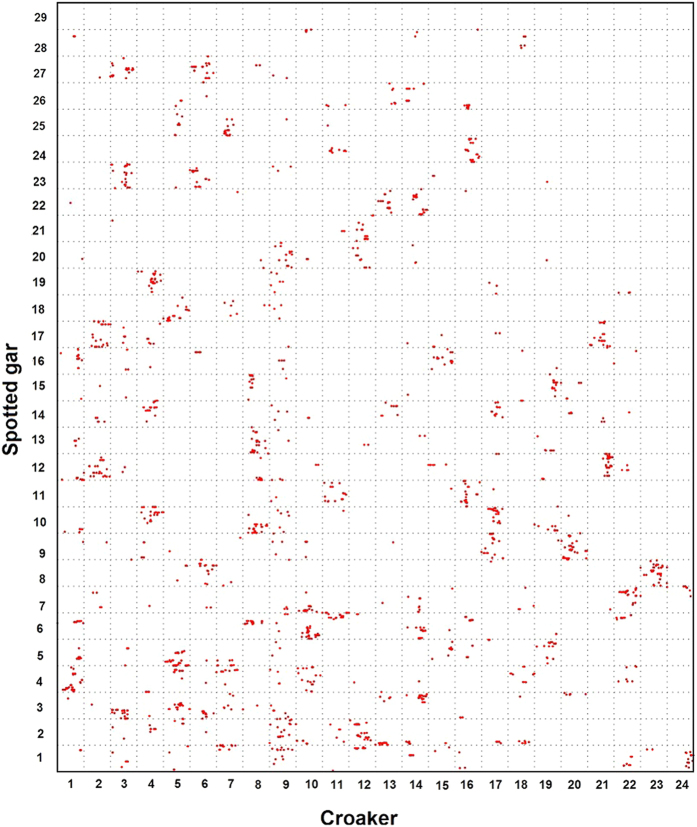
Genomic comparisons between large yellow croaker and spotted gar. In general, 2:1 synteny between croaker and spotted gar was indicated in the Oxford grid.

Finally, we observed two different conservation scenarios between the spotted gar and croaker (Fig. 5). One scenario is that each gar chromosome was generally orthologous to two croaker chromosomes (Fig. 5). For example, gar genes on chromosome 2 had orthologs that were distributed broadly over the two croaker chromosomes, LG9 and LG12. The other scenario is that each portion of a gar chromosome was generally orthologous to parts of two different croaker chromosomes. For instance, gar genes that mapped to the left part of chromosome 10 had orthologs that were distributed broadly over the two croaker chromosomes, LG4 and LG17, whereas genes located on the right portion of chromosome 10 had croaker orthologs that were distributed over LG8 and LG19. Both scenarios revealed 2:1 synteny between croaker and spotted gar. Our observation was consistent with the finding of Amores *et al.*^16^ that each gar chromosome was generally orthologous to two teleost chromosomes. These results supported the notion that teleosts had one more round of whole genome duplication than spotted gar after the speciation event. Due to the small chromosome size of gar chromosome 29 (293.7 kb) and the small number of genes present in the chromosome (only five genes), we did not find gar orthologs to croaker genes in this chromosome. Taken together, the above five comparisons supported that one more round of whole genome duplication occurred in teleosts and that the genome rearrangements in fish differed after they diverged from the ancestor.

### Conserved growth-associated regions and genes

**Figure 6:**
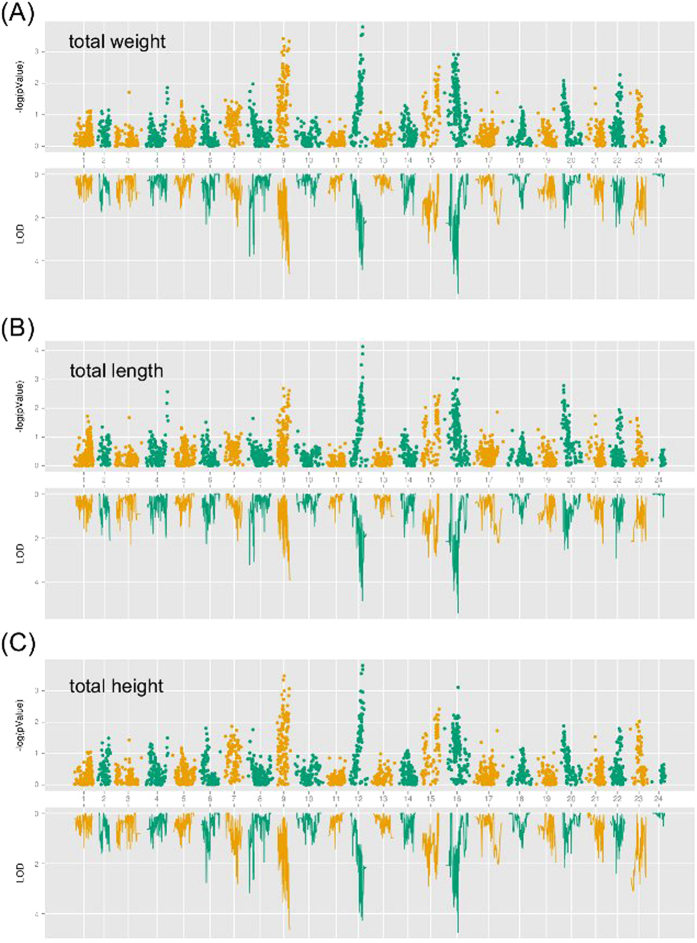
Growth-related association analysis and QTL identification in large yellow croaker. Significantly, loci and regions were identified in the association analysis (up) and in the QTL mapping (down) for total weight (**A**), total height (**B**) and total length (**C**) for all individuals. The significance of each locus is represented as the negative logarithms of the estimated p-value and LOD value.

Pairwise comparisons among growth traits (total weight (TW), total height (TH) and total length (TL)) using Pearson’s correlation revealed that all of the traits were statistically correlated (correlation coefficients ranging from 0.96 to 0.97; see Table 2 for *t*-test p-values). In general, QTL mapping analysis of these growth traits showed that they exhibited quite similar LOD profiles (Figs 6A–C, Table 3, Supplementary Table 4). As a complementary approach, an association analysis revealed a similar distribution pattern across all linkage groups to that found by QTL mapping analysis (Fig. 6), generally supporting the QTL mapping results. Both the QTL analysis and the association analysis suggested that these traits might be regulated by the same set of genes, consistent with the significant correlations among the traits.

In total, three significant QTLs including 86 markers for TW were distributed on croaker LG9, LG12, and LG16 (Fig. 6A and Table 3). Homolog searches identified 41 protein-coding genes in these QTL regions (Fig. 6B and Supplementary Table 5). One major QTL located on LG16 at 45.6 ∼ 74.8 cM presented the highest LOD value of 5.5. The QTL mapping analysis revealed the presence of four significant QTLs containing 56 markers for TL. The location of these QTLs overlapped the significant QTL regions for TW. Twenty-five protein-coding genes were located in these four QTL regions (Fig. 6B and Supplementary Table 5). The major QTL on LG16 at 66.3 ∼ 74.8 cM exhibited the highest LOD value of 5.4. For TH, we identified four significant QTLs including 18 coding genes (Fig. 6C). The QTL regions largely overlapped the regions for TW and TL. The QTL for TH showing the highest LOD value of 4.7 was also identical to the major QTL regions for the previous two traits. Interestingly, 31 markers corresponding to 16 genes were shared among all three traits, consistent with our observation that these traits were highly correlated. The Gene Ontology distributions resembled each other because of the presence of large overlapping QTL regions and genes. Gene Ontology analysis of all associated genes indicated that these genes’ ontologies were widely distributed among the processes of development, cellular component biogenesis and growth (Supplementary Figure 6).

Because the major markers were located in croaker protein genes and protein-coding genes are highly conserved across vertebrates^17^, a knowledge of the functions of orthologs in other species during development and growth would be helpful for understanding the molecular mechanisms of growth-related traits. The participation of orthologs to croaker growth-related genes in muscle development and body growth has been validated. The alpha-adducin (*ADD1*) gene was significantly associated with TW (p-value = 0.001726) and TL (p-value = 0.0008) and was observed in corresponding QTL regions in this study. Functional analysis of this gene showed that *ADD1* was involved in various biological processes that were related to development and growth, including multicellular organism growth (GO:0035264), positive regulation of angiogenesis (GO:0045766), and homeostasis of cell number within a tissue (GO:0048873). In mice, the targeted deletion of *ADD1* resulted in growth retardation at birth and throughout adulthood^18^. Therefore, we speculated that *ADD1* might play a similar function in regulating the development and growth of croaker as its orthologs in mammals.

The beta-catenin (*CTNNB1*) gene was also identified to be associated with croaker TW (p-value = 0.0092) and TL (p-value = 0.0079). The Gene Ontology functions assigned to this gene indicated that it participated widely in development, including liver development (GO:0001889), ectoderm development (GO:0007398), midgut development (GO:0007494), osteoclast differentiation (GO:0030316) and muscle cell differentiation (GO:0042692). Association and QTL analyses in swine indicated that *CTNNB1* was associated with skeletal myogenesis, muscle development and meat quality^19^,^20^,^21^,^22^. We therefore speculated that *CTNNB1* might also regulate body and muscle growth in the croaker.

The triosephosphate isomerase 1 (*TPI1*) gene catalyzes the conversion of dihydroxyacetone phosphate (*DHAP*) to glyceraldehyde-3-phosphate (*G3P*)^23^. This gene was significantly associated with the croaker TL trait (p-value = 0.0094). Previous findings demonstrated that *TPI1* was a key gene in the glycolytic pathway and played an important role in energy generation and muscle cell development^24^. Proteomics and transcriptome analysis showed that *TPI1* protein expression level was positively correlated with growth period in egg- and meat-producing chickens and in pork, and with body size, muscle fat content and growth potential in bulls^25^.

It was also suggested that orthologs of other associated croaker genes, including heat shock 70 kDa protein 5 (*HSPA5*) and apolipoprotein Eb (*APOEB*), regulated growth and metabolism in other species^26^. The participation of orthologs to croaker genes in growth and development in other vertebrates is consistent with our observation of the significant association between these genes and croaker traits. The identified growth-related genes and markers in the croaker may be useful for selective breeding of croaker.

## Discussion

Genetic maps are among the most important genomic resources for the genetic and evolution study of species^27^ and widely used for genome assembly, chromosome construction and functional gene mapping^16^. Although croaker genetic maps based on several hundreds of markers featuring amplified fragment length polymorphisms (AFLPs) and simple sequence repeats (SSRs) were reported^28^,^29^, the lack of a high-density linkage map hampered both the genome-wide comparative analysis and the detailed mapping of functional regions representing economic traits. In this study, a linkage map containing 3,448 markers was constructed for the croaker. To our knowledge, this is the first high-density linkage map reported for the croaker. The average resolution of this linkage map was 0.76 cM, and 2,495 of the markers in the linkage map resided in protein coding regions. Our map provided sufficient resolution for fine-scale QTL mapping and facilitated the discovery of quantitative trait genes. Furthermore, the high proportion of protein-coding markers enabled us to perform a chromosome-level comparative analysis.

The error rate of this map was estimated to be very low (1.8%), and the quality of this map was sufficiently high to conduct downstream analysis. We further examined the reason for the erroneous distribution of genome sequences to different groups. The first possible reason is the existence of cross-over events across progeny. We successfully PCR amplified eleven sequences across four samples (Supplementary Figure 4). We found that the mapped markers in these eleven sequences were fully covered by RNA-sequencing reads from all samples (Supplementary Figure 7). These two results suggested that no cross-over event occurred in these loci. The second possible reason is inaccurate variant calling. We estimated that the false positive rate of variant calling in our study was 2.8%. The third possible cause of the erroneous linkage grouping of markers might be the use of Joinmap. Wu *et al.* reported that Joinmap might erroneously group markers^30^. Regardless of the possible reasons for the errors, the very low rate at which they occurred demonstrated the high quality of this map.

**Figure 7:**
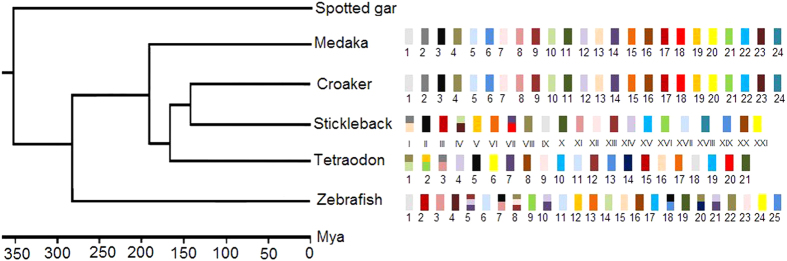
Teleost genome rearrangements. Phylogenetic relationships among spotted gar, croaker and other four species were constructed based on the results of Amores *et al.*^16^ and Wu *et al.*^8^. The figure illustrates genome rearrangements in the medaka, croaker, stickleback, tetraodon and zebrafish genomes. In croaker, twenty-four linkage groups are represented by the differently colored bars. In other species, the chromosomes have the same colors as those corresponding to orthologous croaker chromosomes.

The common ancestor of teleost species experienced three rounds of whole-genome duplication^31^. Previous studies reported that many teleosts underwent inter-chromosomal rearrangements after speciation from a common ancestor^4^,^7^. In the present study, we performed an *in silico* analysis of orthologous gene pairs to study genome-wide synteny and rearrangements across six species. First, the comparison illustrates the conservation scenario that the croaker preserves the ancestral karyotype, as medaka has. Protein sequence comparison showed that stickleback was phylogenetically related more closely to the croaker than to other teleosts^8^,^9^. However, almost all croaker and medaka chromosomes exhibit 1:1 correspondence, whereas three stickleback chromosomes have 1:2 syntenic correspondences to croaker linkage groups. This finding suggests that the karyotype of the croaker is closer to that of the medaka than to that of the stickleback. Because the medaka preserves its ancestral karyotype, the low level of evidence of inter-chromosomal rearrangements between the two species indicates that the karyotype of the croaker might also be similar to that of the ancestor. Second, the comparisons reveal wide and different rearrangement events in teleosts other than the medaka and croaker. In both the stickleback and tetraodon, three chromosomes have 1:2 syntenic correspondences to croaker linkage groups. However, these correspondences occurred in different croaker chromosomes, suggesting different genome fusion events in the stickleback and tetraodon. The genome rearrangements in zebrafish are more complex than those in the stickleback and tetraodon. The comparison reveals 1:2 and 1:3 syntenic correspondences between zebrafish and the croaker (Fig. 4). Figure 7 summarizes the possible genome rearrangements in five teleost species.

In this study, the generation of a high-resolution genetic map allowed us to perform QTL fine mapping of the growth traits of the croaker. Growth traits are of interest to breeding researchers due to their high commercial significance in aquaculture^32^,^33^,^34^. QTL mapping represents an efficient approach to the identification of genetic loci underlying these traits for marker-assisted selection in genetic breeding^35^. Four shared growth-related QTLs were detected in the linkage groups LG9, LG12 and LG16 and were supported by the association analysis. Gene Ontology analysis provides hints that the genes in these four QTLs participate in growth, further supporting the QTL mapping results. The markers located at the confidence intervals of these QTLs constitute a valuable marker set for the further evaluation of the utility of these markers in marker-assisted selection. The discovery of growth trait genes demonstrates the potential of our map for application into other important croaker traits.

In conclusion, we applied RNA-seq technology to the large-scale identification of SNPs that were then successfully used for high-throughput genotyping and construction of a high-resolution croaker genetic map. The obtained genetic map is the most comprehensive genetic map to date for this commercially important species. With this map, we identified syntenic relationships among the croaker, medaka, stickleback, tetraodon, zebrafish and spotted gar using comparative genomic analysis. Through mapping analysis and an association study, we identified four significantly overlapping QTL regions for TW, TH and TL that will aid the achievement of breeding goals for croakers. The large numbers of generated SNPs and the dense genetic map should not only lay a foundation for chromosomal-level analysis of the croaker genome but also should provide an excellent resource for future molecular breeding efforts, such as genomic selection. Our SNP-based genetic map will be applied to an update of the croaker genome sequence, which will be published in the near future.

## Methods

### Ethics Statement

The study and all experimental protocols were approved by the Animal Care and Use Committee of the Fisheries College of Jimei University. The methods were carried out in accordance with approved guidelines.

### Animals and sequencing

Seventy-two F1 adult individuals of one full-sib family of croakers were selected for genotyping. Growth-related traits, including TW, TH, and TL, were measured for all progeny. DNA was extracted from the dorsal fins of two parents with a TIANamp Genomic DNA Kit (TIANGEN, Beijing, China). The purified genomic DNA was fragmented by ultrasound, and libraries with insert lengths of approximately 300 bp were constructed in a similar manner to that described in previously published protocols^8^,^9^. The Illumina HiSeq 2000 platform was used for pair-end (2 × 100 nt) sequencing. Genomic sequencing reads were archived in the Sequence Read Archive (SRA) database (project accession no. PRJNA278908).

For each F1 individual, tissue samples were excised from liver, spleen, kidney, gonad, heart and brain tissues. Total RNA was isolated and purified from all six tissues using TRIzol (Invitrogen, Carlsbad, CA, USA) following the manufacturer’s instructions. The extracted RNA was incubated with DNase I (Takara, Dalian, China) for 1 h at 37 °C to eliminate contaminating genomic DNA. Only RNAs with RNA integrity number (RIN) values of over 8 (tested with a Bioanalyzer 2100 instrument; Agilent Technologies, Palo Alto, CA, USA) were retained for downstream RNA-seq library construction. Library construction and transcriptome sequencing were performed according to the Illumina RNA-seq protocol. The libraries were sequenced on an Illumina HiSeq 2000 platform (Illumina, Inc., San Diego, CA, USA), and read lengths of 2 × 100 nt were obtained.

### Genome assembly and genotyping

First, the raw reads were filtered to remove ambiguous or low-quality reads using the HTQC package^36^. Clean genomic reads from the male parent were assembled using SOAPdenovo (default parameters)^37^. To decrease heterozygous genomic regions, the sequences assembled using SOAPdenovo were further assembled using CAP3 software (default parameters)^38^. The CAP3 sequences were used as the reference genome for further genotyping.

Genomic sequencing reads of two parents and the RNA-seq reads of F1 progeny were aligned to the reference genome using CLC genomic workbench (CLC Bio). To ensure accurate alignment, the mapping processes allowed a maximum of two gaps or mismatches for each read. We discarded reads that were mapped to multiple regions. Then, SNP identification was performed using a quality-based strategy with the following parameters: (1) the minimum average quality of surrounding bases and the minimum quality score of the central base were 15 and 20, respectively; (2) a sequencing depth was greater than 6. Genotyping data that were homogeneous in both parents or missing in more than 20% of the offspring were removed. Genotypes in individuals violating Mendel’s law of segregation were eliminated.

### Map construction

Markers that segregated and could be genotyped in at least 80% of the individuals were considered to be high-quality markers and were retained for further analysis. For each segregating marker, we performed a chi-square test to identify markers conforming to the expected Mendelian ratio (p-value > 0.05). The identified markers were included in map construction using JoinMap4.0^14^. Because JoinMap is unable to process more than 5,500 markers, computational time was reduced by retaining only the marker with the highest coverage among all individuals during downstream linkage map construction if multiple markers with identical genotypes were present in a single genomic sequence. The linkages between markers, recombination rate and map distances were calculated using JoinMap’s population type CP (cross pollinator, or full-sib family), the Kosambi mapping function^39^ and the regression mapping algorithm. Markers were grouped at a logarithm of odds (LOD) threshold of at least 5.0.

### SNP validation

Although the high sequencing depth and stringent filtration criteria would minimize the false positive variants obtained from RNA-sequencing data, alignment errors and inaccurate base calling might still result in putative uncertainties in variant calling. Therefore, true positive rates and false positive rates were used in this study to estimate the accuracy of variant calling by comparing SNPs from Sanger sequencing to those from RNA-sequencing for the same regions of the same individuals.

To assess the reliability of SNP calling from RNA-sequencing, potentially erroneously grouped SNPs in eleven genomic sequences were amplified and validated by PCR analysis using DNA from four mapped individuals. All primers used for PCR amplification and SNP validation are listed in Supplementary Table 2. The resulting PCR products were sequenced by an ABI 3730 xl genetic analyzer and standard protocols. For each region, only parts with good-quality Sanger sequence data of each individual were used for the estimation of accuracy rate; consequently, the lengths of the sequences ranged from 150 to 500 bp. To identify SNPs, the Sanger sequences were aligned against the reference genome sequences using CLUSTALW^40^. By comparing variants based on Sanger and RNA-sequencing data, a SNP was called to be true positive when it was detected by both methods.

### Comparative genomes and naming

Because the markers were identified by RNA-seq data, they very likely represent coding sequences. Blastx^15^ searches were conducted against proteins from the stickleback, medaka, zebrafish, tetraodon and spotted gar (downloaded from the Ensembl^41^ database) with an e-value cutoff of 1e^−5^ and allowing the association of croaker coding contigs to highly similar annotated genes of these five genomes. For the sequences without homologs in the above model fish genomes, to identify more protein-coding genes, we further aligned them against the NCBI nr database using Blastx^15^ with an e-value cutoff of 1e^−5^.

To facilitate further comparative analysis with the medaka genome, we ordered the linkage groups based on marker similarity to medaka genes. The best-aligned hit gene in the medaka genome was selected for each marker. If most markers in a group were linked to a medaka chromosome, the linkage group was named with the medaka chromosome number. We constructed Oxford grids^42^ by aligning all croaker coding markers according to their genomic order in each linkage group on the horizontal axis and then plotted the position of orthologs in other species genomes on the vertical axis. The genome-wide comparisons between croaker and other species were performed following the above strategy.

### QTL mapping analysis and association study of growth traits

Pairwise correlations among three growth traits were performed across all progeny using Pearson correlations. Assuming that the correlation of two random traits in the mapping family was equal to zero, we tested whether the correlation of two traits was statistically significant by comparison to zero with *Student’s t*-test (p-value ≤ 0.05).

QTL mapping analysis was performed for three growth traits using MapQTL 6 software (http://www.kyazma.nl/index.php/mc.MapQTL). Composite interval mapping^43^ and multiple QTL model (MQM) mapping^44^ were utilized to detect significant markers associated with the traits of TW, TL and TH. LOD score significance thresholds were calculated by 1,000-permutation tests and a linkage group-wide significance level of α < 0.05.

As a complementary approach to QTL mapping, association analysis was performed between genotypes and growth traits using Plink 1.07^45^, which performs a simple linear regression of phenotype on genotype. Markers with p-values ≤ 0.01 were considered significantly associated with growth traits. To identify whether the orthologs of these significantly associated loci were also associated with growth in other species, we aligned the candidate genomic scaffolds in QTL regions against the NCBI nr database by Blastx^15^. GO annotation of the aligned protein-coding genes was performed with NCBI2R (http://ncbi2r.wordpress.com/) as implemented in the R package^46^.

## Additional Information

**How to cite this article**: Xiao, S. *et al.* Gene map of large yellow croaker (*Larimichthys crocea*) provides insights into teleost genome evolution and conserved regions associated with growth. *Sci. Rep.* **5**, 18661; doi: 10.1038/srep18661 (2015).

## Supplementary Material

Supplementary Information

Supplementary Table 1

Supplementary Table 2

Supplementary Table 3

Supplementary Table 4

Supplementary Table 5

## Figures and Tables

**Table 1:** Summary of the consensus linkage map in large yellow croaker.

linkage group	length (cM)	marker number	number of annotated markers	average marker interval (cM)
1	102.5	194	123	0.52
2	67.8	90	63	0.75
3	143.8	168	114	0.85
4	119.1	175	113	0.68
5	124.0	221	152	0.56
6	110.0	147	104	0.74
7	98.0	123	80	0.79
8	136.1	263	166	0.51
9	71.2	138	112	0.51
10	151.3	218	125	0.69
11	96.2	121	79	0.79
12	94.4	126	93	0.75
13	123.8	104	77	1.2
14	94.9	202	139	0.47
15	99.8	85	55	1.17
16	136.0	191	110	0.71
17	158.4	180	127	0.88
18	130.2	104	56	1.25
19	109.9	127	80	0.86
20	111.2	119	81	0.93
21	101.8	106	67	0.96
22	85.6	131	92	0.65
23	92.3	80	40	1.15
24	72.7	35	22	2.08
total	2,632.0	3,448	2,270	0.76

**Table 2:** Correlation analysis of three growth traits of large yellow croaker.

correlation coefficient	TW	TL	TH
TW	1		
TL	0.967 (p < 2.2e-16)	1	
TH	0.973 (p < 2.2e-16)	0.959 (p < 2.2e-16)	1

**Table 3:** Genomic regions associated with growth traits using QTL mapping in large yellow croaker.

traits	linkage groups	region (cM)	LOD	marker number
TW	9	61.8 ∼ 71.2	4.6	3
	12	54.6 ∼ 69.2	4.4	21
	16	45.6 ∼ 74.8	5.5	62
TL	9	71.1 ∼ 71.2	3.9	1
	12	52.6 ∼ 71.5	4.9	23
	16	47.6 ∼ 52.6	4.4	7
	16	66.3 ∼ 74.8	5.4	25
TH	9	67.1 ∼ 71.2	4.6	1
	12	52.1 ∼ 69.2	4.2	23
	16	50.1 ∼ 50.2	3.7	1
	16	69.5 ∼ 74.8	4.7	11
