# Supplementary material for: Gene map of large yellow croaker (Larimichthys crocea) provides insights into teleost genome evolution and conserved regions associated with growth
Source: Sci Rep. 2015 Dec 22;5:18661. doi: 10.1038/srep18661 (PMC4687042; doi:10.1038/srep18661)
Supplement: Supplementary Information [file srep18661-s1.doc]

SUPPLEMENTARY INFORMATION FOR

Gene map of large yellow croaker (*Larimichthys crocea*) provides insights into teleost genome evolution and conserved regions associated with growth

Shijun Xiao^1^, Panpan Wang^1^, Yan Zhang^2^, Lujing Fang^1^, Yang Liu^1^, Jiong-Tang Li^2,*^ and Zhi-Yong Wang^1,*^

**Affiliations**

^1^ Key Laboratory of Healthy Mariculture for the East China Sea, Ministry of Agriculture, P.R.China; Fishery College, Jimei University, Yindou Road, Xiamen, P.R. China

^2^ CAFS Key Laboratory of Aquatic Genomics and Beijing Key Laboratory of Fishery Biotechnology, Centre for Applied Aquatic Genomics, Chinese Academy of Fishery Sciences, Beijing, 100141, P.R. China

* Correspondence and requests for materials should be addressed to J.T.L. ([lijt@cafs.ac.cn](../../../../迅雷下载/lijt@cafs.ac.cn)) and Z.Y.W ([zywang@jmu.edu.cn](mailto:zywang@jmu.edu.cn))

**Supplementary Figures:**

**Supplementary Figure 1: Average RNA-sequencing depth for markers in 72 offsprings .**

We calculated the average sequencing depth for all 23,790 markers passing SNPs and genotype filtering for each individual. The error bars represent the standard variances among those markers.

**
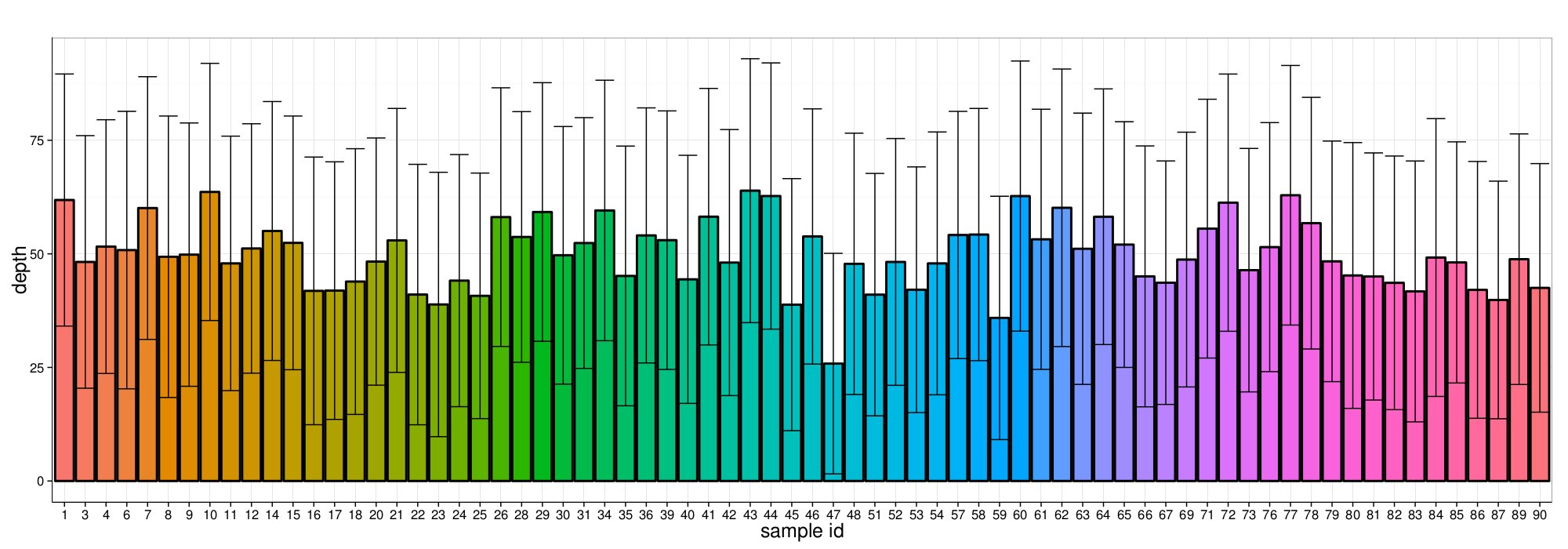
**

**Supplementary Figure 2: Linkage groups of croaker.**

Markers are listed on both sides of the linkage groups. Marker name is composed of scaffold id and base pair location, separated by an underline.


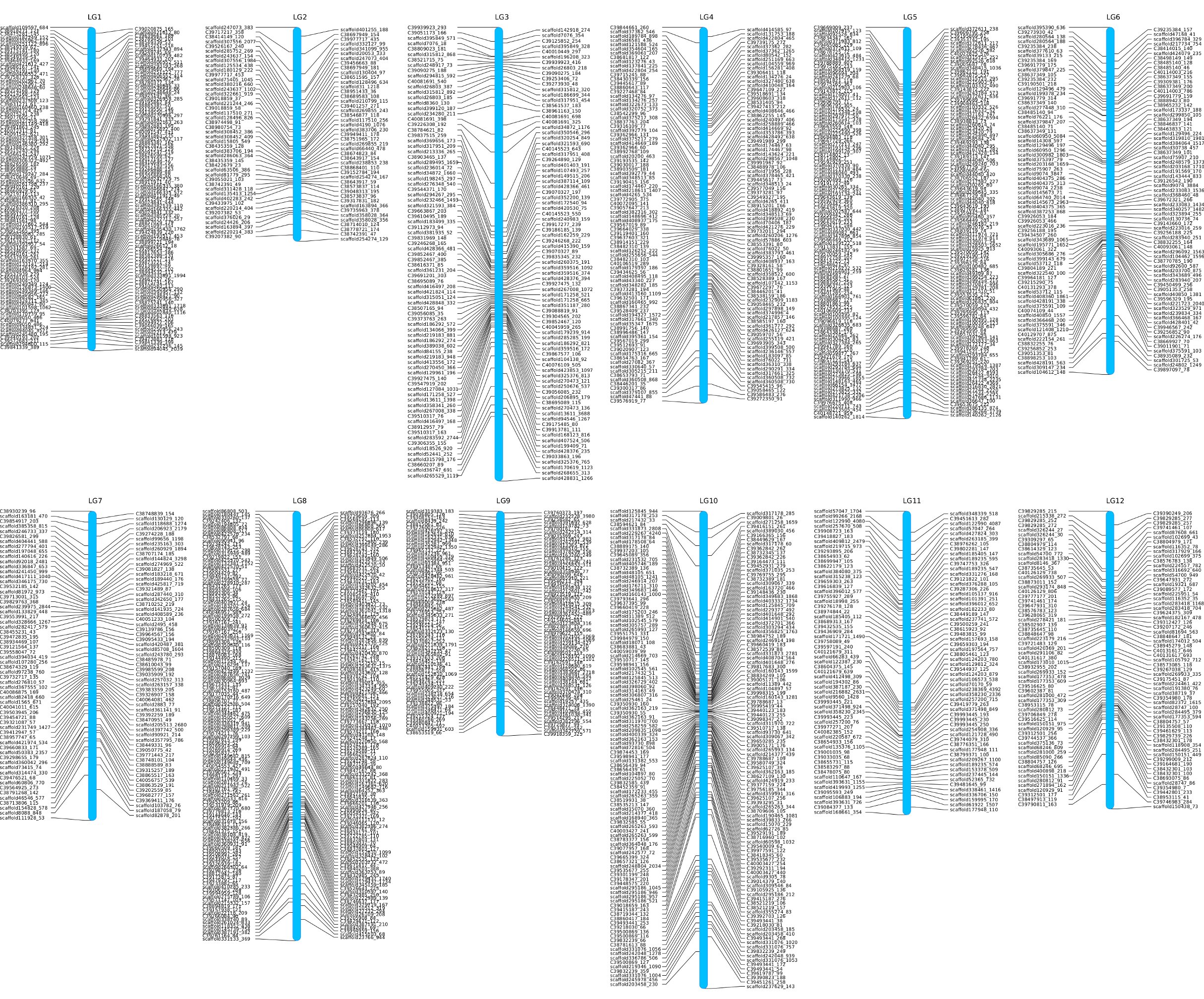


**
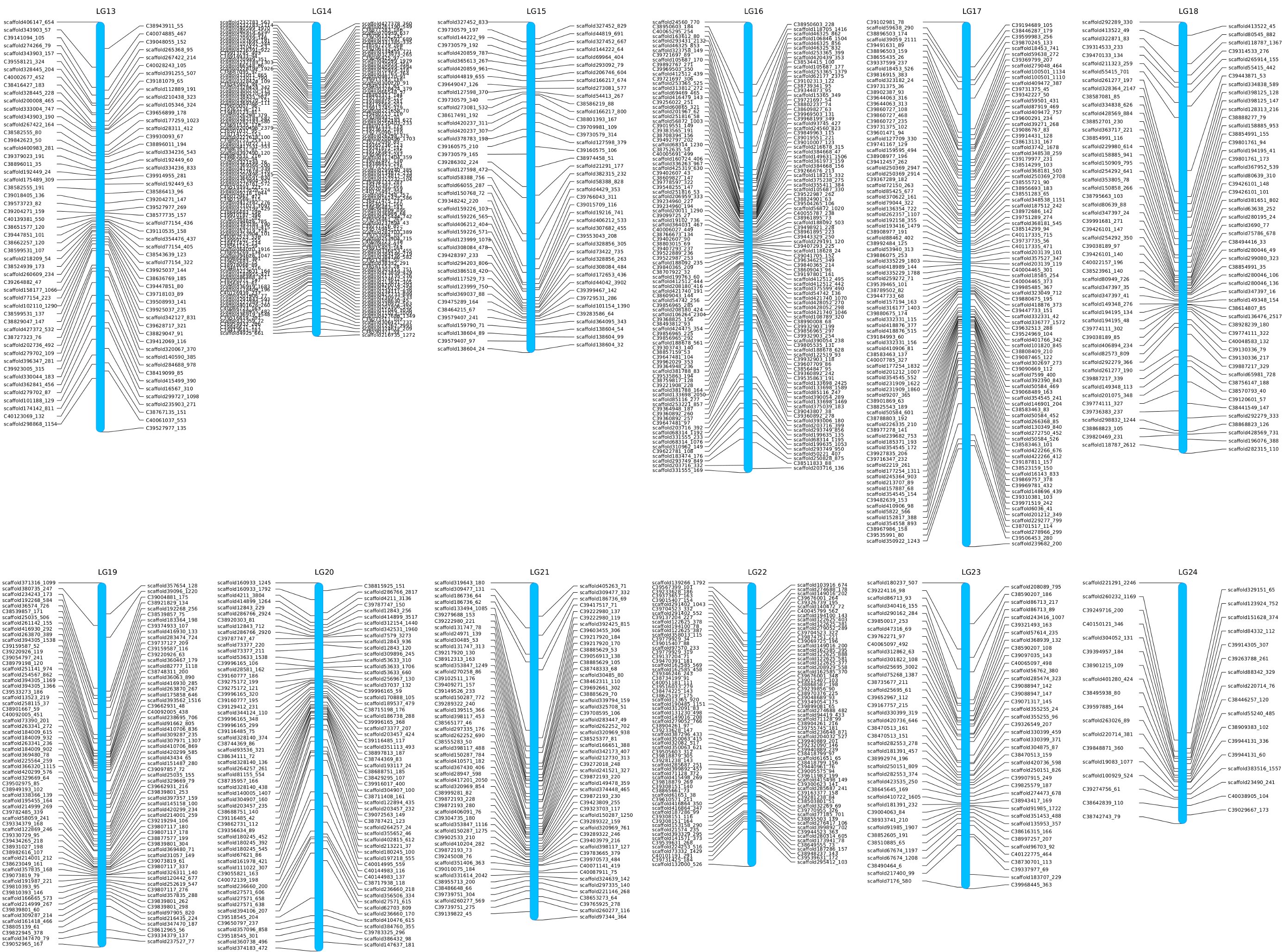
**

**Supplementary Figure 3: Length distribution of marker scaffolds.**

Sequence number count (Y-axis) is plotted against each length interval (X-axis). Note that the last bar accumulates all sequences longer than 3k bp.


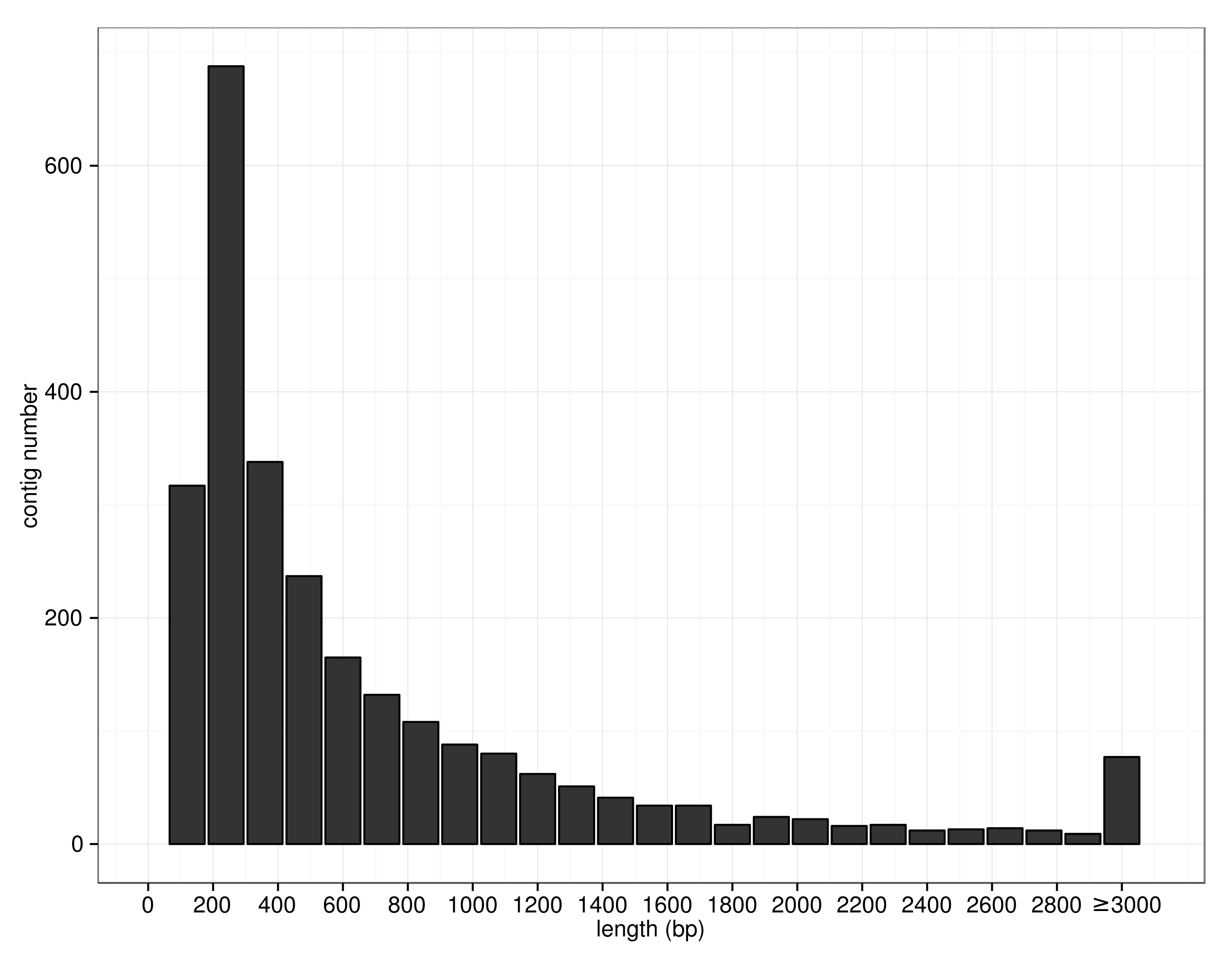


**Supplementary Figure 4: the assembled sequences validated by PCR.**

Eleven reference sequences with suspicious markers were PCR amplified in four samples (father, sample 44, sample72, and sample77). The primers were listed in Supplementary Table 2. M stands for Markers. Sample 131 represents father sample.


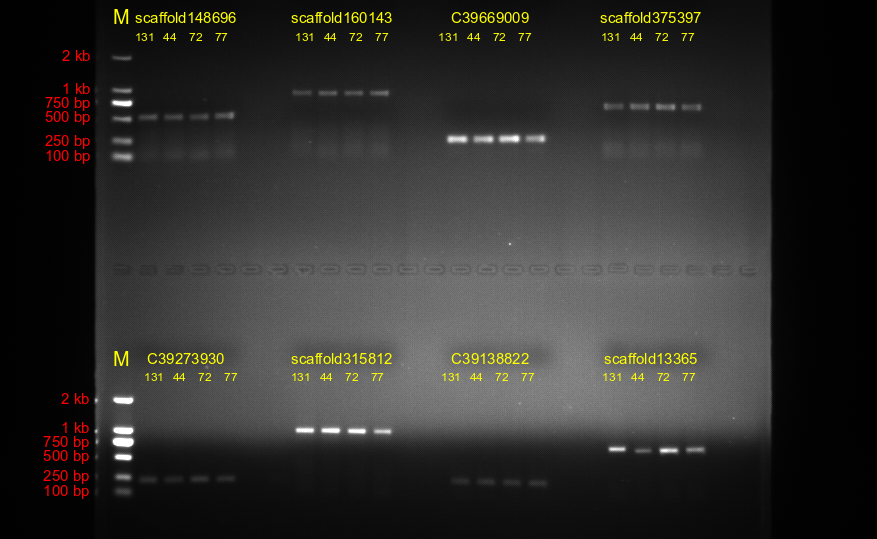

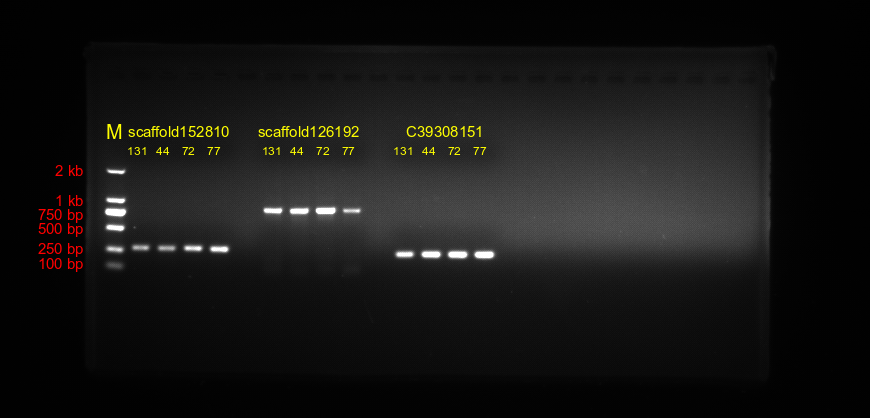


**Supplementary Figure 5: SNP validation by Sanger sequencing.**

A total of 36 regions correspond to nine markers (C39273930_42, C39138822_224, C39308151_116, C39308151_140, C39308151_164, C39669009_237, C39669009_238, scaffold315812_892 and scaffold315812_868) in four samples (father, sample 44, sample72, and sample77) were selected for Sanger sequencing to validate genotypes. The genotypes called by CLC and sample names were shown alongside the arrows. The arrows indicated the Sanger sequencing results.

**
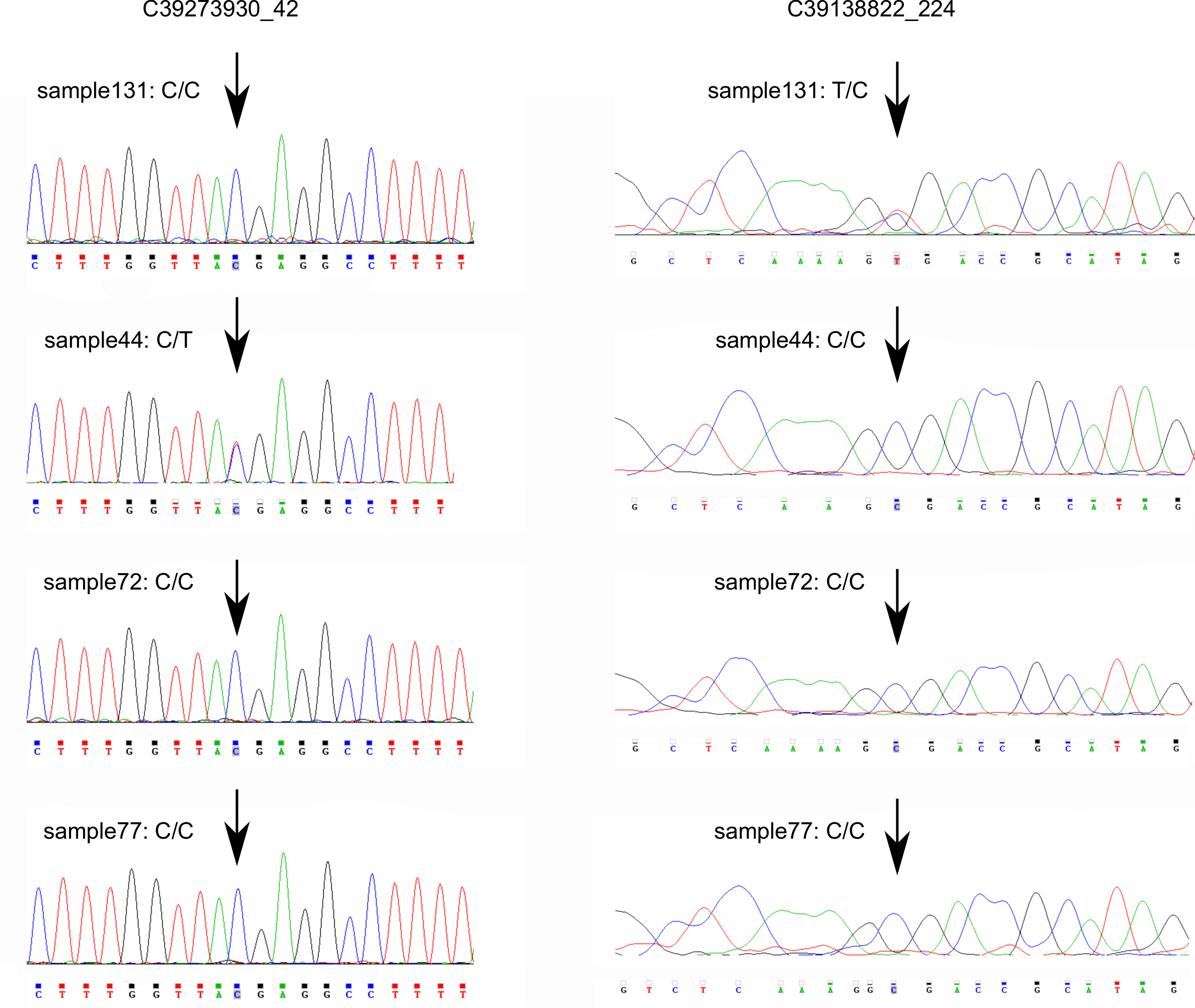
**

**
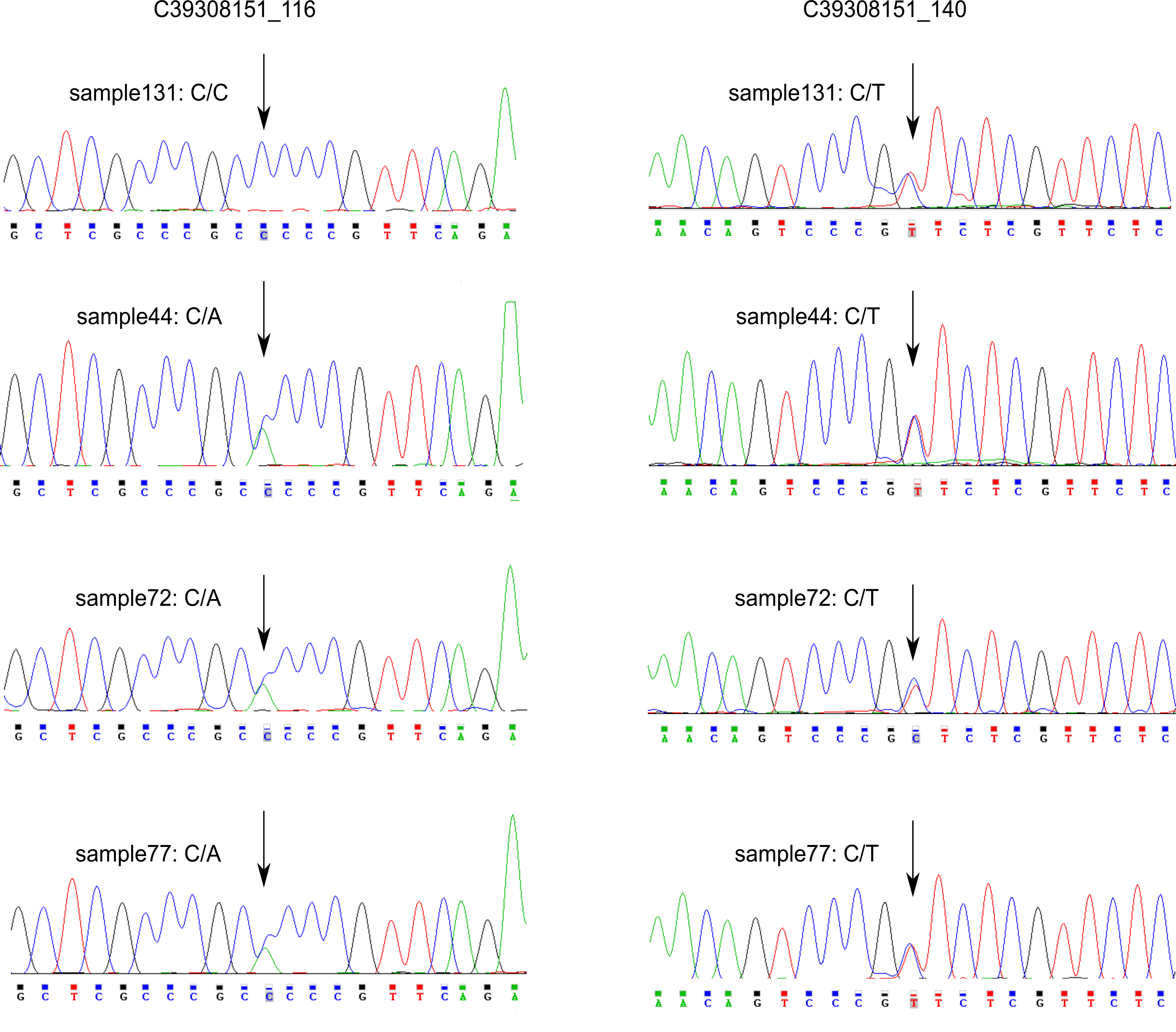
**

**
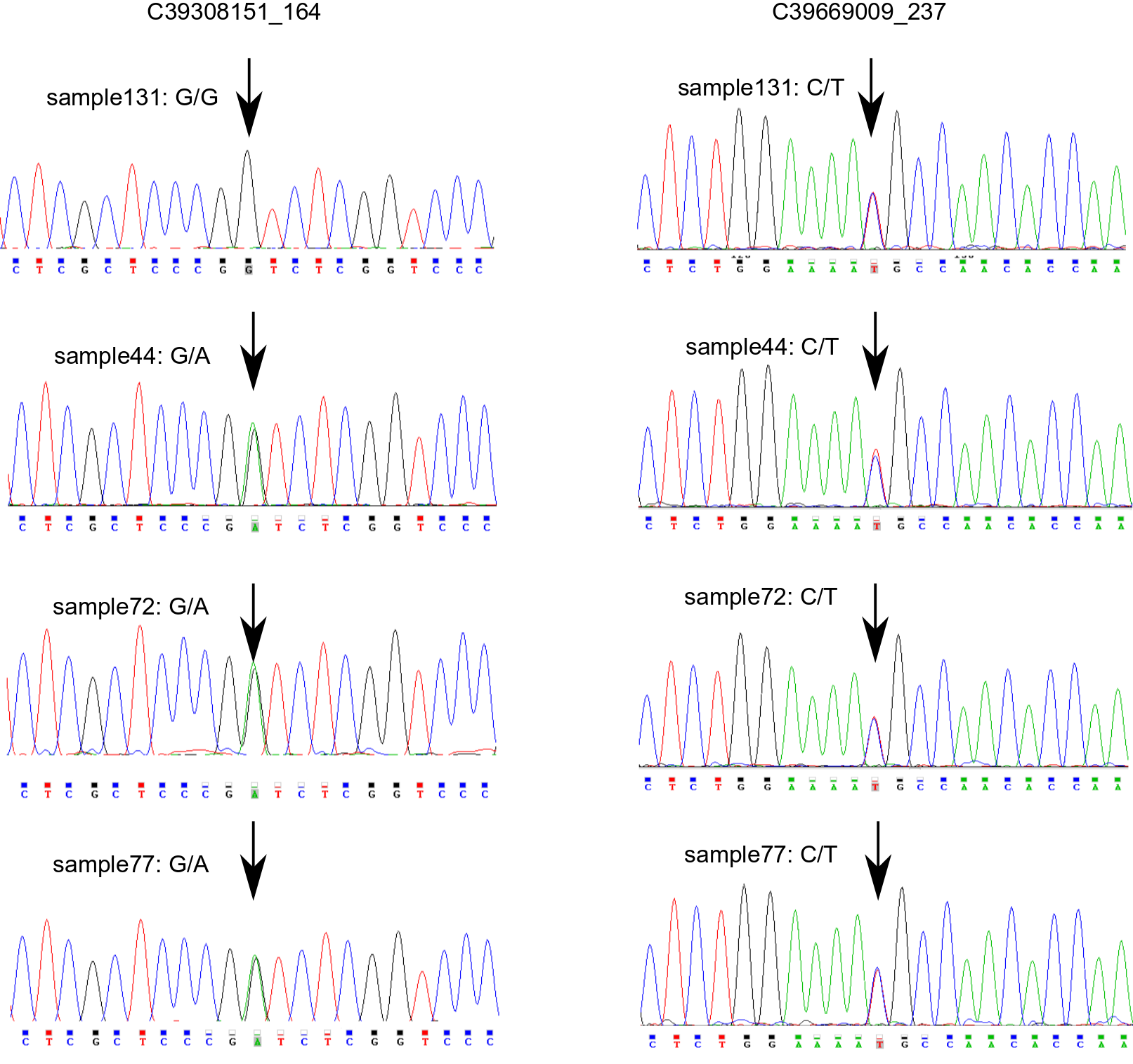
**

**
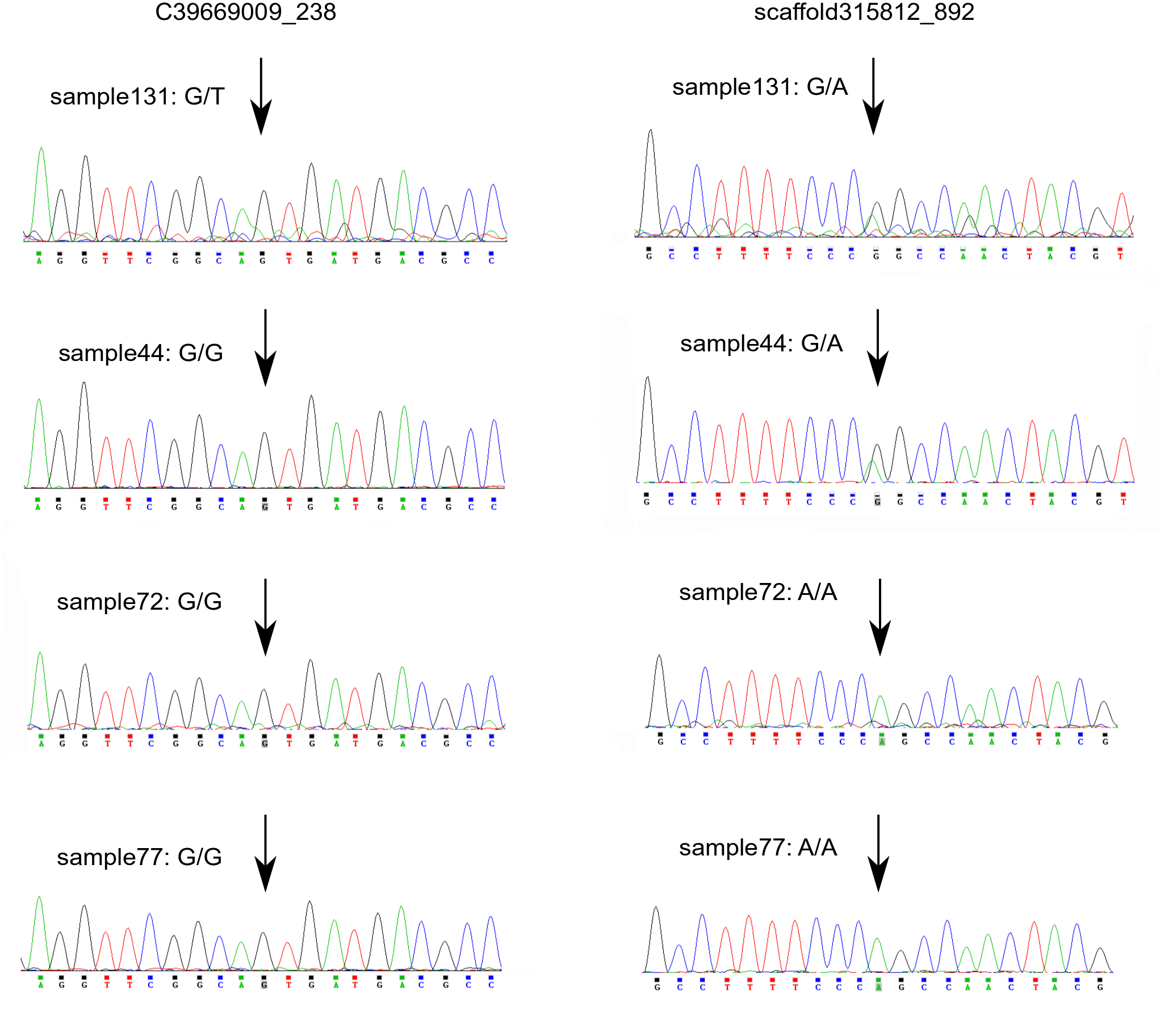
**

**
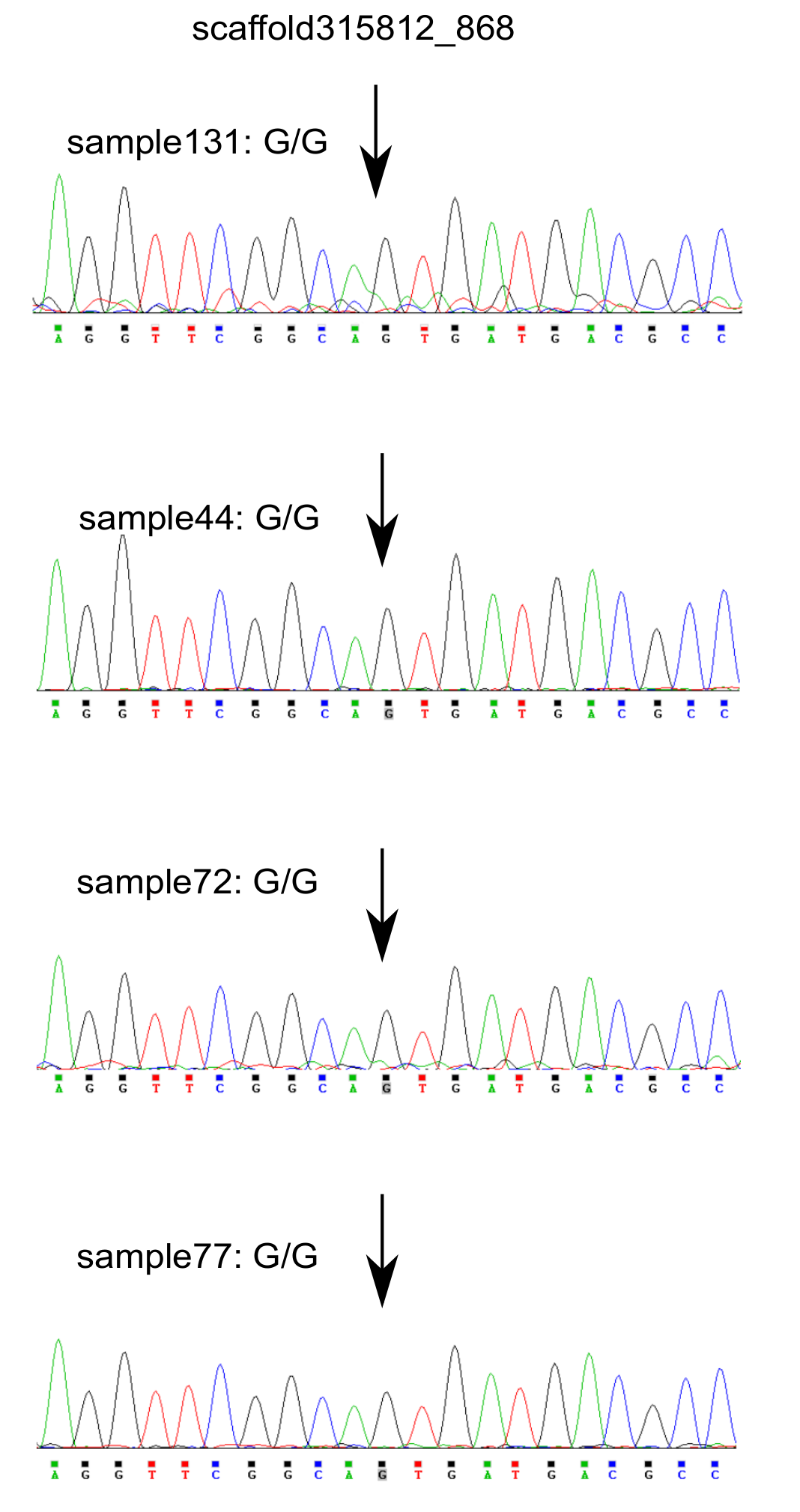
**

**Supplementary Figure 6: Venn diagram and functional annotation of associated markers and genes for three growth traits.**

Venn diagram illuminated a large portion of associated marker (A) and genes (B) were shared in total weight (blue), total length (orange) and total height (green). (C)The percentage of associated genes (Y-axis) was plotted against with each GO annotation level (X-axis) using WEGO[^1^](#_ENREF_1).
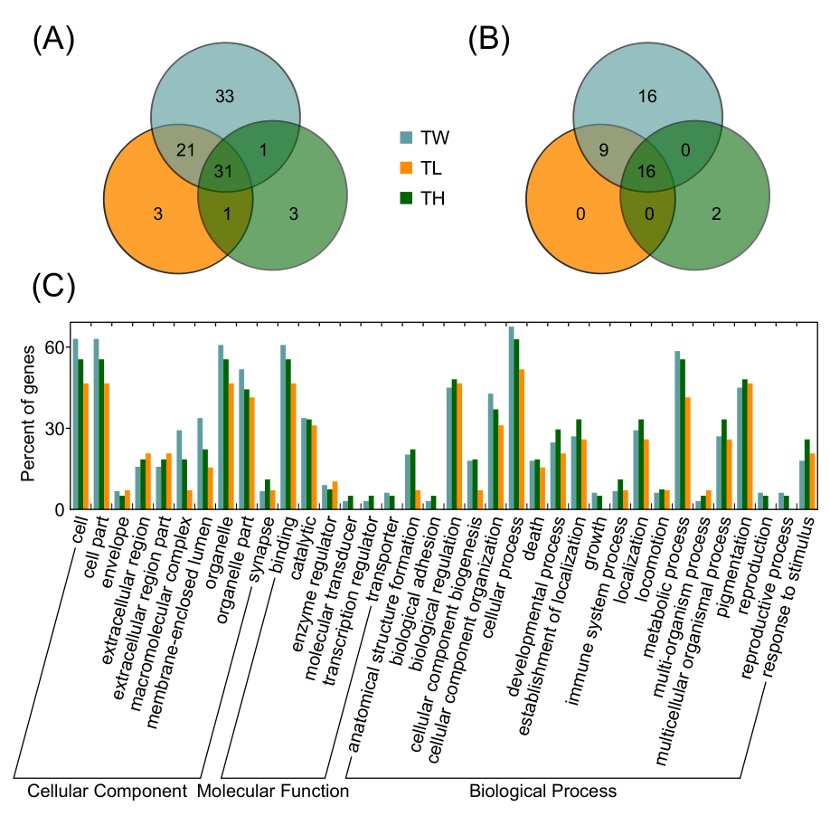


**Supplementary Figure 7: reads alignment to 11 reference sequences with suspicious SNP markers.**

The suspicious SNP markers were shown at the bottom of figures. The bases of A, T, C, and G were shown in green, red, blue, and brown, respectively. For each marker, we selected one read covering it to represent each individual. We found that the mapped markers in these 11 sequences were fully covered by RNA-sequencing reads from all samples, indicating no cross-over events in these sequences.

C39138822:


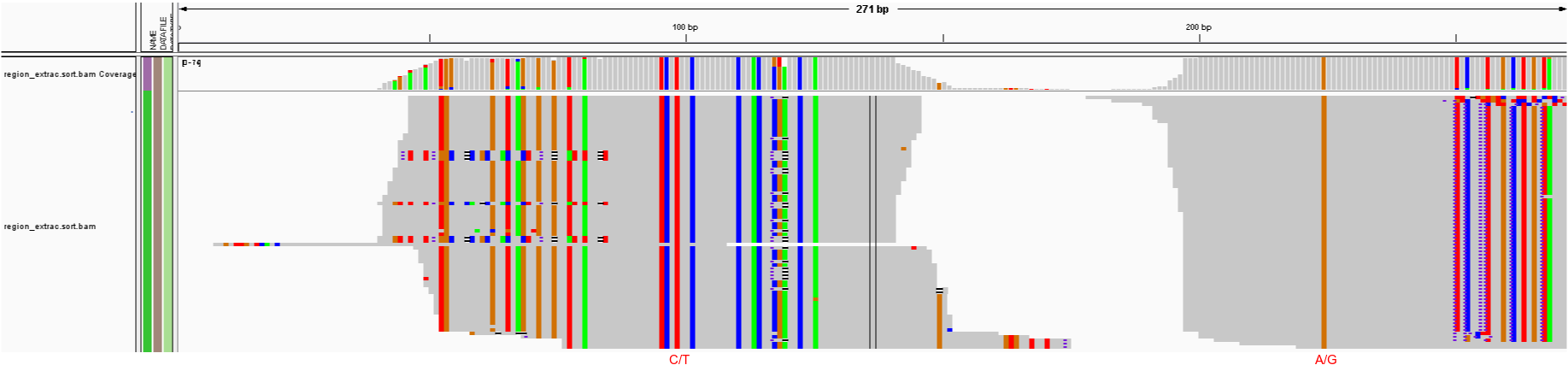


C39273930

**
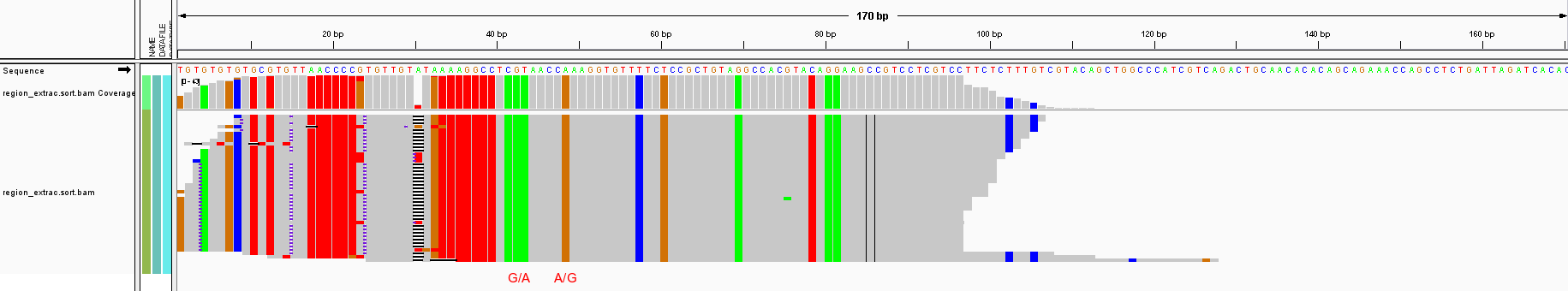
**

C39308151


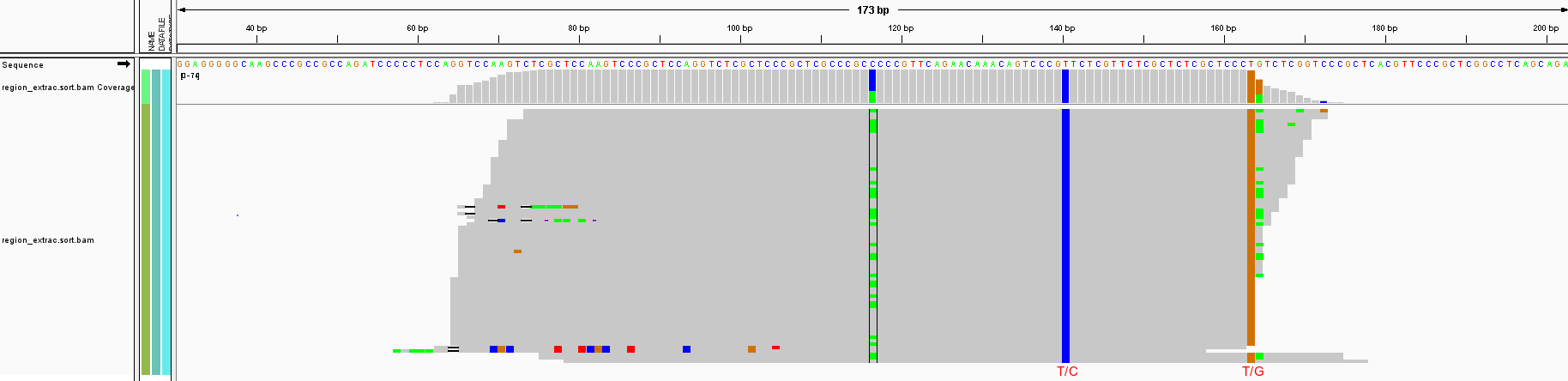


C39669009


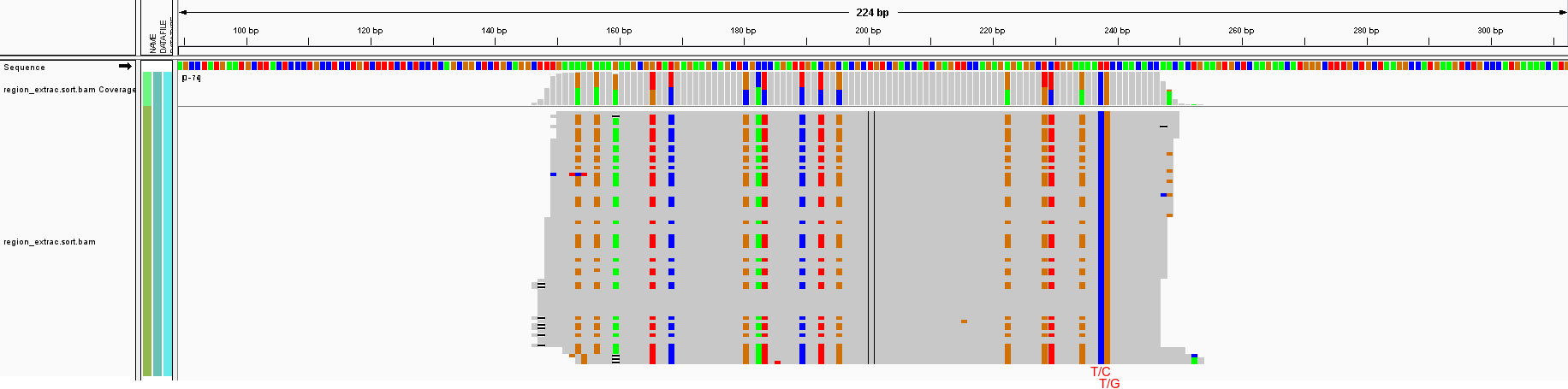


scaffold13365


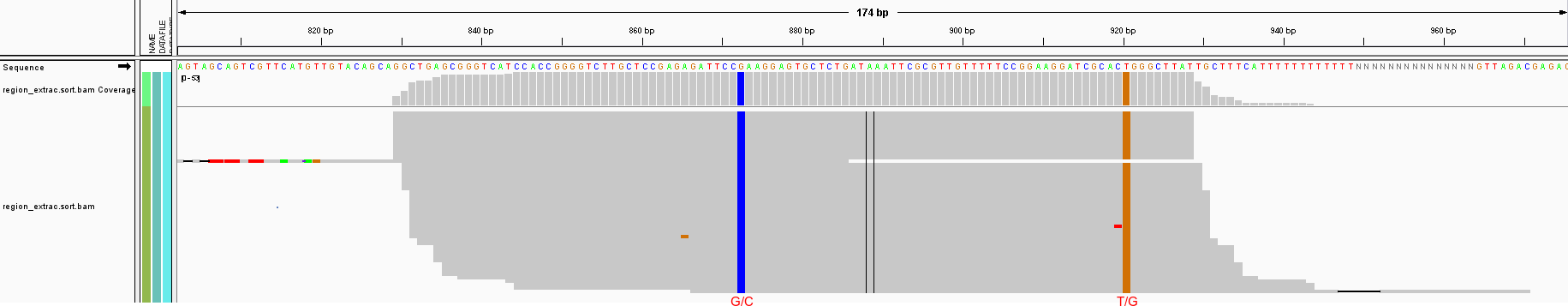


scaffold126192


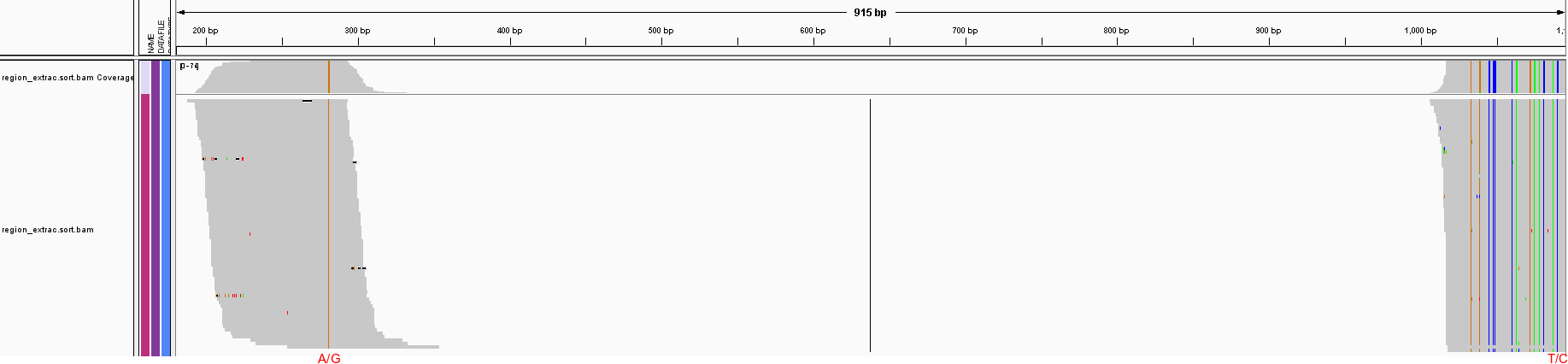


scaffold148696


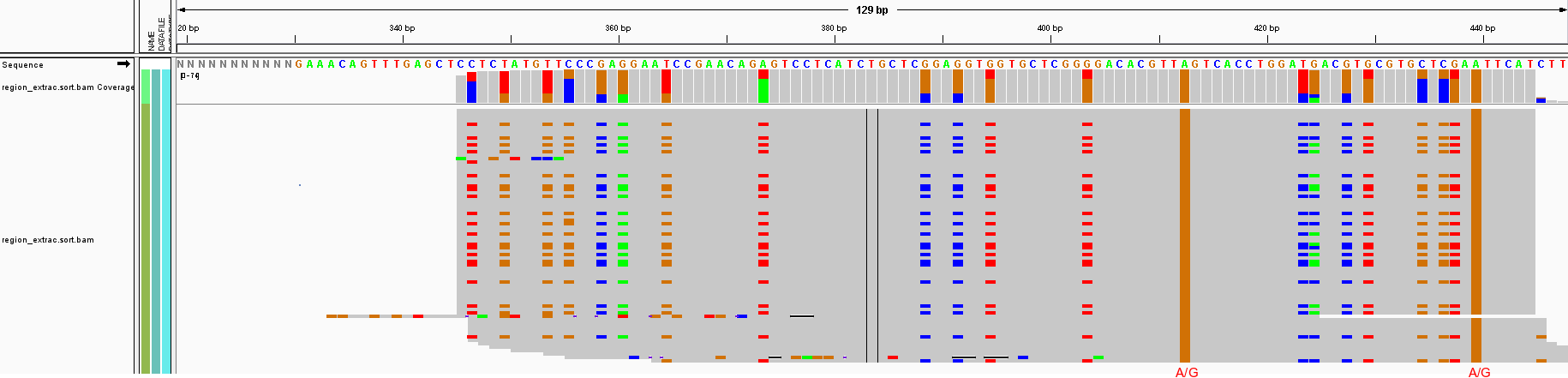


scaffold152810


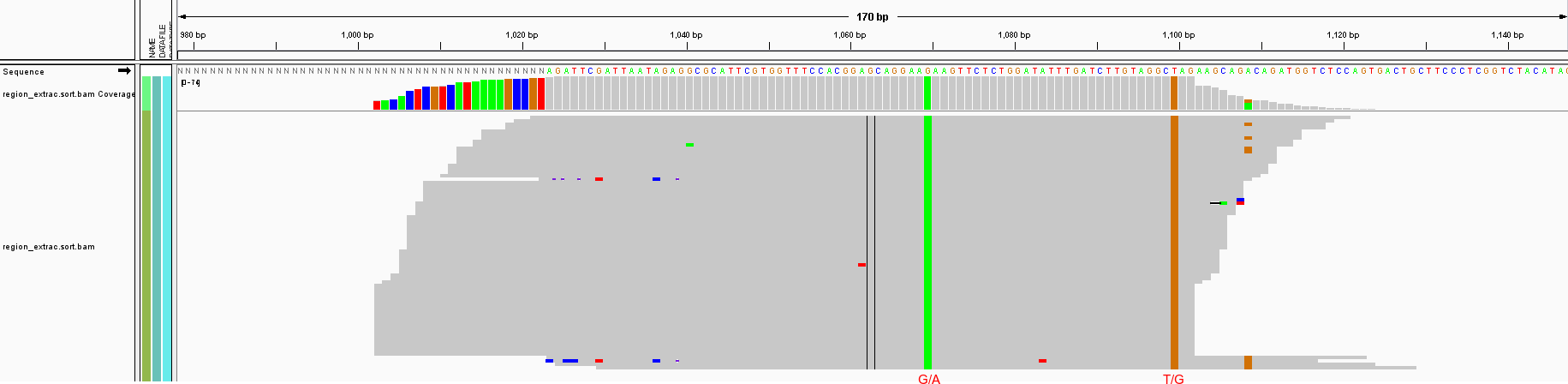


scaffold160143


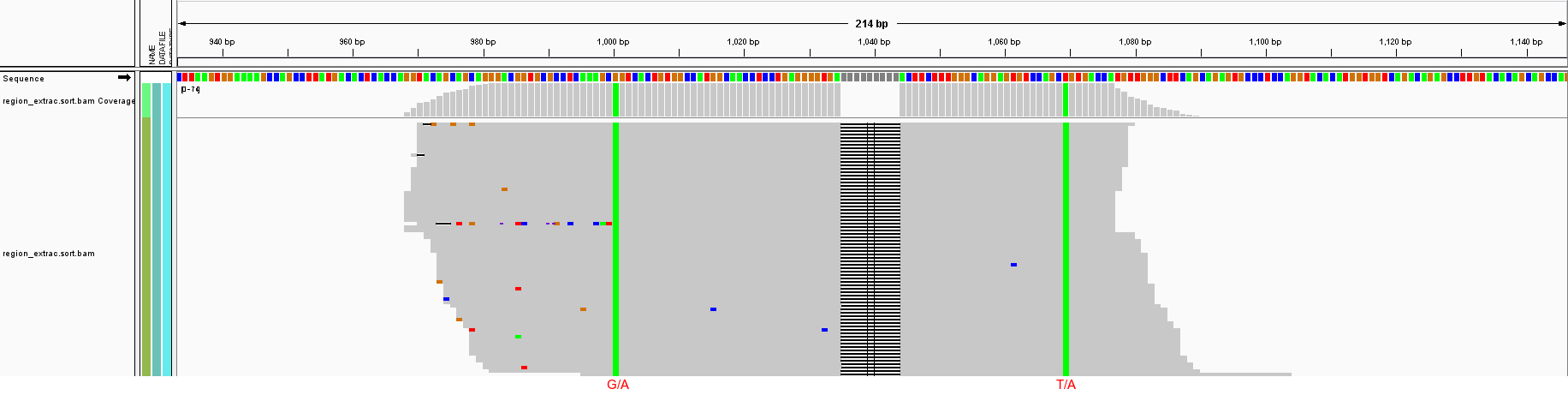


scaffold315812


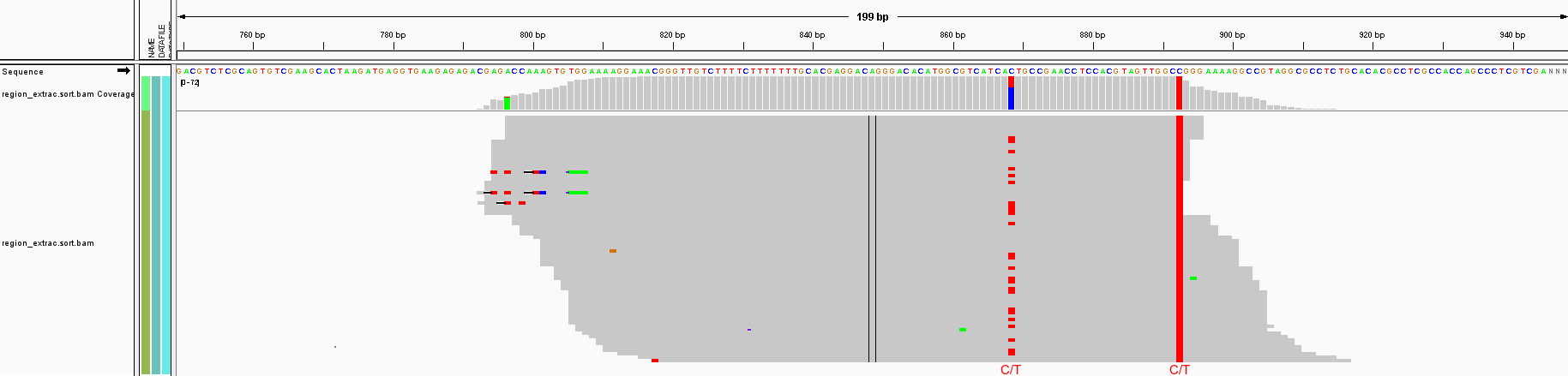


scaffold375397


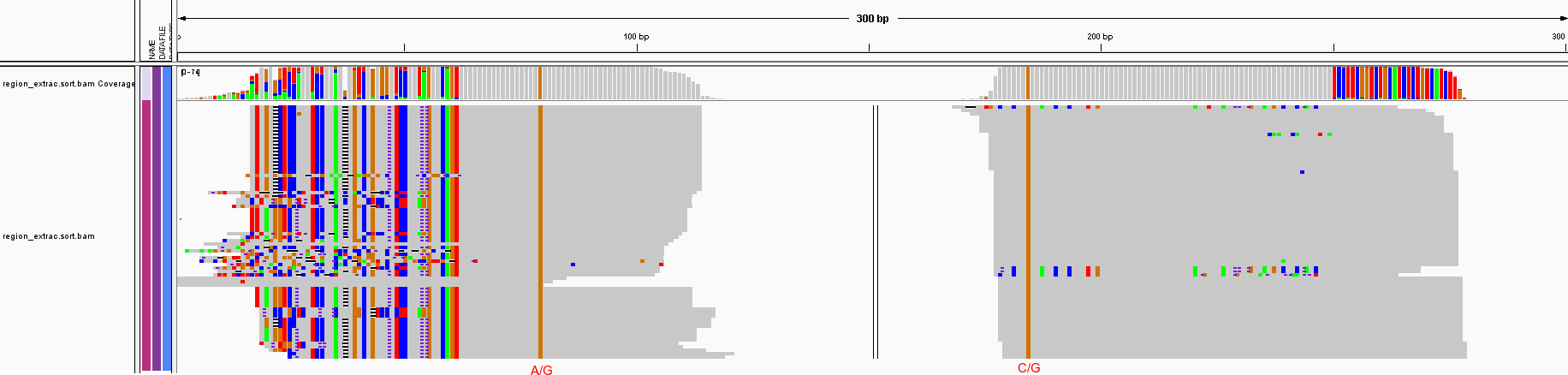


**Supplementary Tables:**

**Supplementary Table 1: Linkage group number and marker location in croaker.**

(Included in a separated excel file)

**Supplementary Table 2: Primers used for reference sequence PCR amplification.**

(Included in a separated excel file)

**Supplementary Table 3: Ortholog correspondence of croaker to five model fish species and the NCBI nr database.**

(Included in a separated excel file)

**Supplementary Table 4:** **Genomic region and genetic markers associated with croaker growth traits.**

(Included in a separated excel file)

**Supplementary Table 5: Gene location and GO annotation linked with associated markers to total weight (TW), total length (TL) and total height (TH).**

(Included in a separated excel file)

**Reference**

1 Ye, *J. et a*l. WEGO: a web tool for plotting GO annotations*. Nucleic Acids Resear*c**h** 34, W293-W297, doi:10.1093/nar/gkl031 (2006).
